# Clinical Evaluation of the Turkish Version of the Eating Behavior after Bariatric Surgery (EBBS) Scale and its Association with Postoperative Weight Outcomes

**DOI:** 10.1007/s11695-026-08633-1

**Published:** 2026-03-27

**Authors:** Inci Kirtil, Yeşim Di̇kmen Aydin, Zeliha Özlem Akoğlu, Cihan Şahan

**Affiliations:** 1 Department of Nursing, Yeditepe University, Istanbul, Turkey; 2 Department of Nursing, Marmara University, Istanbul, Turkey; 3 Sultan 2. Abdülhamid Han Eğitim ve Araştırma Hastanesi, Istanbul, Turkey; 4 Aktif International Hospital, Istanbul, Turkey

**Keywords:** Obesity, Metabolic bariatric surgery, Eating behavior, Clinical applicability

## Abstract

**Introduction:**

Long-term weight loss after metabolic bariatric surgery largely depends on patients’ adherence to postoperative dietary and lifestyle recommendations, yet tools to clinically assess such adherence across different cultures remain limited. The Eating Behavior after Bariatric Surgery scale is a contemporary measurement tool developed to assess postoperative behavioral patterns, including nutrition-related behaviors, self-monitoring, and physical activity. This study aimed to translate this scale into Turkish and to explore its clinical relevance by examining associations with postoperative weight-related outcomes.

**Methods:**

This cross-sectional study included 298 patients who had undergone metabolic bariatric surgery. Sociodemographic and surgical characteristics, pre- and postoperative weight measures, and subscale scores were collected. The translation and cultural adaptation of the scale involved forward–back translation and expert review, without formal psychometric validation. Relationships between the subscale scores and weight-related outcomes were examined using correlation analyses and multiple linear regression to evaluate clinical relevance.

**Results:**

Mean total weight loss was 33.5 ± 16.2 kg. The subscale scores indicated highest adherence for drink consumption (5.3 ± 0.9) and lowest for general behaviors (3.3 ± 1.4). Correlation analyses showed significant associations between the subscales and weight-related outcomes. In regression analysis, preoperative weight and time since surgery positively predicted postoperative total weight loss percentage, whereas food consumption adherence was negatively associated with postoperative total weight loss percentage (R²=0.245; *p* < 0.01).

**Conclusion:**

In this study, higher adherence scores in the food consumption domain were unexpectedly associated with lower postoperative total weight loss, highlighting the complex relationship between self-reported eating behaviors and weight outcomes. These findings suggest that the Turkish version of the scale may provide clinically informative insights into postoperative eating behaviors, while highlighting the complex relationship between self-reported adherence and weight outcomes.

**Key Points:**

The Turkish version of the EBBS enables a structured clinical assessment of postoperative eating and lifestyle behaviors in patients who have undergone metabolic bariatric surgery.

EBBS domain scores demonstrated domain-specific associations with postoperative weight-related outcomes, supporting their clinical relevance rather than formal psychometric validity.

Higher self-reported adherence in the food consumption domain was unexpectedly associated with lower postoperative total weight loss, underscoring the complex and non-linear relationship between self-reported eating behaviors and weight outcomes after surgery.

## Introduction

Obesity is currently recognized as a chronic health problem with a rapidly increasing prevalence worldwide [1, 2]. Medical problems associated with obesity, such as hypertension, diabetes, cardiovascular diseases, and certain types of cancer, increase mortality rates and significantly impair individuals’ quality of life [3]. According to the Turkish Health Survey 2022, the prevalence of obesity among individuals aged 15 years and older in Türkiye was 20.2%, with higher rates observed in women (23.6%) than in men (16.8%). In addition, 30.9% of women and 40.4% of men were classified as overweight [4]. Although national registry data reporting the annual number of metabolic bariatric surgery (MBS) procedures in Türkiye are not publicly available, this substantial proportion of individuals with obesity and pre-obesity underscores the growing demand for effective metabolic management strategies, including MBS for eligible patients.

MBS is considered an effective treatment option for individuals with severe obesity, as it promotes clinically meaningful weight loss, metabolic improvement, and enhanced quality of life [5, 6]. Nevertheless, postoperative weight trajectories vary considerably among individuals. Weight loss and long-term weight maintenance after MBS are influenced by a complex interplay of biological mechanisms, such as hormonal and metabolic adaptations, behavioral factors including dietary practices and physical activity, and broader psychosocial and environmental influences, rather than by a single isolated determinant [7, 8].

Weight loss and maintenance following MBS depend not only on the surgical intervention itself but also on the continuation of healthy eating behaviors and lifestyle modifications in the postoperative period [9]. Inadequate adherence to postoperative dietary and lifestyle recommendations has been associated with nutritional deficiencies, suboptimal weight loss, and weight regain in some patients [10, 11]. Postoperative eating behaviors and lifestyle patterns may change over time and can contribute, alongside biological and psychosocial factors, to variability in weight outcomes and quality of life [12, 13]. Accordingly, structured postoperative follow-up delivered by a multidisciplinary team is considered essential for monitoring dietary intake, physical activity, and broader lifestyle behaviors in patients who have undergone MBS [12, 14].

The Eating Behavior after Bariatric Surgery (EBBS) questionnaire is a self-report instrument developed to assess postoperative eating and lifestyle behaviors across four domains: food consumption, drink consumption, general eating behaviors, and lifestyle behaviors [10]. Previous studies evaluating the EBBS has demonstrated its utility in capturing multidimensional behavioral patterns relevant to the postoperative period [10, 11]. In the original development study, EBBS scores were examined in relation to postoperative weight-related parameters, providing insight into behavioral adherence patterns following surgery [10]. Subsequent cross-cultural adaptation research has further explored associations between EBBS domain scores and postoperative weight outcomes, suggesting that the questionnaire may offer clinically informative perspectives on postoperative behavioral adjustment, although the performance of behavioral constructs may vary across cultural contexts [11]. In previous studies, EBBS scores were examined in relation to postoperative weight-related parameters, providing insight into behavioral adherence patterns following surgery. Specifically, the researchers found that the Lifestyle domain (particularly regular physical activity and weekly self-weighing) was the most significant predictor of weight loss maintenance. Additionally, avoiding carbonated beverages was found to be a key virtuous behavior for maintaining weight stability [10, 11].

Given the cultural dietary patterns, social eating practices, and lifestyle characteristics specific to Türkiye, examining postoperative behavioral adherence within this context is particularly relevant. Behavioral responses to MBS are known to be context-dependent, and differences in food choices, beverage consumption, and physical activity patterns may influence how self-reported behavioral measures relate to clinical outcomes. Therefore, the present study was not designed to replicate the original psychometric validation of the EBBS. Instead, it aimed to translate the EBBS into Turkish and to examine its clinical applicability and contextual relevance by exploring associations between EBBS domain scores and postoperative weight-related outcomes in patients who have undergone MBS.

## Methods

This study was conducted with patients who underwent surgery at the MBS clinic of a private hospital in Istanbul between June and August 2025. Participants were recruited using a non-probability convenience sampling method. Inclusion criteria were being 18 years of age or older, volunteering to participate in the study, having undergone surgery at least six months prior, and having no communication barriers. For the purpose of this study, “communication barriers” referred to conditions that could interfere with the participant’s ability to understand and accurately complete a self-report questionnaire, including insufficient proficiency in Turkish, severe cognitive impairment, or acute psychiatric conditions. Postoperative care at the study center follows standard clinical practice and typically includes routine surgical follow-up visits and nutritional counseling as part of multidisciplinary bariatric care. However, detailed individual-level data regarding the frequency, duration, or type of postoperative support (e.g., dietitian or psychological counseling) were not systematically collected in this study and therefore were not included in the analyses.

The sample size was determined based on the number of eligible patients who met the inclusion criteria and consented to participate during the study period. Accordingly, this study was conducted with a total of 298 participants who met the specified sampling criteria. Research data were obtained using the Personal Information Form, developed by the researchers in line with the literature, and the EBBS scale.

### Personal Information Form

Developed based on relevant literature [10, 11, 15], this form consisted of questions designed to capture participants’ sociodemographic and descriptive characteristics (age, gender, date of surgery, type of surgical procedure, height, preoperative weight, lowest postoperative weight achieved, current weight, and postoperative total weight loss [TWL]).

### Eating Behavior After Bariatric Surgery (EBBS)

The EBBS is a self-administered questionnaire developed to assess patients’ adherence to recommended dietary and lifestyle behaviors after MBS. The scale consists of 11 items grouped into four subdomains. The food consumption subscale evaluates meal frequency, intake of sweets, and portion size of vegetables. The drink consumption subscale assesses fluid intake during meals, alcohol consumption, and consumption of carbonated beverages. The general behaviors subscale captures eating-related behaviors such as eating speed, perceived hunger before meals, and satiety after meals. The lifestyle subscale evaluates physical activity frequency and self-monitoring behaviors, including regular self-weighing. Each item is scored on a scale from 0 to 2, with responses categorized and structured according to predefined criteria. The EBBS consists of four subscales with different numbers of items and score ranges. The food consumption subscale includes 5 items, with total scores ranging from 0 to 10. The drink consumption subscale comprises 4 items, with possible scores ranging from 0 to 8. The general behaviors subscale consists of 4 items, with total scores ranging from 0 to 8. The lifestyle subscale includes 3 items, with possible scores ranging from 0 to 6. The total score is calculated by summing the responses to all items, with higher scores indicating greater adherence to postoperative recommendations [10].

### Cross-Cultural Adaptation

The translation of the EBBS into Turkish was informed by commonly cited recommendations for the cross-cultural adaptation of self-report measures. The process included forward translation by bilingual translators, reconciliation of the translated versions, back translation into the source language, and expert review to ensure semantic and conceptual consistency. These procedures were implemented to enhance linguistic clarity and contextual appropriateness of the Turkish version rather than to establish formal psychometric equivalence. The translation process was guided by the general principles outlined for cross-cultural adaptation of self-report instruments [16].

## Translation and Back-Translation Process

The original English version of the scale was independently translated into Turkish by three translators who were native English speakers, fluent in Turkish, and experienced in the grammatical and cultural characteristics of both languages. The translators carried out this process independently, without prior discussion or collaboration. The resulting Turkish translations were then consolidated, common expressions were identified, and discrepancies were discussed until consensus was reached, leading to a unified draft version. Subsequently, the Turkish version was back-translated into English by a bilingual individual whose native language was Turkish, who had advanced proficiency in English, and who had not previously seen the original scale. This back-translated version was reviewed by another academic and compared with the original form. Necessary revisions were made, and consensus was reached, thereby completing the language validity phase of the adaptation.

## Content Validity

### Expert Opinion

The draft version of the Turkish EBBS was reviewed by subject matter experts to assess item clarity, cultural appropriateness, and clinical interpretability. Expert feedback was collected using a structured rating form based on the Davis technique, in which each item was rated on a 4-point scale ranging from 1 (not relevant) to 4 (highly relevant). Agreement among experts was summarized descriptively to guide item revision rather than to establish formal content validity. Expert input was obtained from a panel of 13 specialists, including academics with expertise in surgical nursing and scale development, dietitian academics specializing in postoperative bariatric nutrition, and experienced bariatric surgeons. Items that received lower relevance ratings (items 2, 5, 7, 8, 9, and 10) were reviewed and revised based on expert suggestions to improve clarity and contextual fit. The revised version was subsequently reviewed by language experts to ensure linguistic accuracy.

## Pilot Testing and Data Collection

The study was conducted in two phases. In the first phase, the draft Turkish version of the scale was pilot-tested with 20 patients to assess item clarity, comprehensibility, and feasibility of administration. Participants were asked to provide feedback on the wording and understandability of the items. No major comprehension difficulties were reported, and no item revisions were required based on this feedback. As the pilot phase did not result in changes to the wording or structure of the questionnaire, responses from these participants were retained in the dataset to maximize sample size. In the second phase, patients who had undergone MBS at least six months prior were informed about the study and invited to participate. All eligible patients were approached. The final sample consisted of 298 participants who completed the scale and a sociodemographic information form. Data collection in the main phase was conducted using a secure online survey platform (Google Forms). No personally identifiable information was collected, and all responses were recorded anonymously.

### Weight-related Outcome Variables

Weight-related outcome variables were defined and calculated in accordance with the original development study of the EBBS scale [10]. Pre-surgery weight (kg) was defined as the body weight measured prior to MBS. Minimum weight (kg) was defined as the lowest body weight reached after the bariatric procedure. Current weight (kg) was obtained by direct measurement at the time of the study. Weight lost (kg) was calculated as pre-surgery weight − current weight. Weight lost (%) was calculated as (pre-surgery weight − current weight) × 100 / pre-surgery weight. Weight lost maintained (%) was calculated as (pre-surgery weight − current weight) / (pre-surgery weight − minimum weight) × 100. Recovery from minimum weight (kg) was calculated as current weight − minimum weight. For descriptive and comparative purposes, participants were additionally categorized according to postoperative weight-related outcomes (e.g., %TWL, weight loss maintenance, and postoperative weight regain). Cut-off values for these categories were determined based on the median or mean values of the study sample, consistent with the analytical approach used in previous EBBS validation studies [11].

### Statistical Analysis

Statistical analyses were conducted using IBM SPSS Statistics version 25. Descriptive statistics were used to summarize participant characteristics, EBBS subscale scores, and weight-related outcomes. EBBS subscale scores were reported both as raw mean values (± standard deviation) and as percentages of the maximum possible score to facilitate interpretation. Categorical variables were presented as frequencies and percentages, while continuous variables were summarized using mean±standard deviation or minimum-maximum values. The normality of continuous variables was assessed using the Kolmogorov-Smirnov test and visual inspection of histograms. Associations between EBBS subscale scores (food consumption, drink consumption, general eating behaviors, and lifestyle behaviors) and weight-related outcomes (total weight loss [kg], percentage of TWL, weight loss maintenance percentage, and weight regain from minimum weight) were examined using Pearson correlation analysis. Correlation coefficients (r) were interpreted based according to Cohen’s criteria as small (0.10–0.29), moderate (0.30–0.49), or strong (≥ 0.50). To evaluate the effect of the time elapsed since surgery on behavioral adherence, participants were divided into three groups based on follow-up duration: early postoperative (< 6 months), mid-term (7–12 months), and late postoperative (≥ 13 months). The distribution of EBBS subscale scores among these groups was compared using one-way analysis of variance (ANOVA), and post-hoc tests were applied to determine specific group differences.

To further explore the clinical relevance of EBBS subscale scores, multiple linear regression analysis was conducted with %TWL as the dependent variable. Independent variables included EBBS subscale scores and clinically relevant covariates, including age, gender, surgery type, preoperative body weight, and time elapsed since surgery. Assumptions of linearity, normality, homoscedasticity, and multicollinearity were evaluated and met prior to analysis. In all statistical tests, significance was set at a two-tailed and p-value of < 0.05.

## Ethical Considerations

Permission to conduct the cross-cultural adaptation of the scale was obtained via email from the corresponding author of the original scale development team. Prior to initiating the study, ethical approval was obtained from the Non-Interventional Clinical Research Ethics Committee of Marmara University (dated 22.05.2025, approval number 88), and institutional permission was secured from the private healthcare facility where the research was conducted. Written and electronic informed consent was obtained from all patients who agreed to participate in the study. All procedures involving human participants were conducted in accordance with institutional and/or national ethical guidelines and the principles of the Declaration of Helsinki.

## Results

### Participant Characteristics, Weight-Related Outcomes, and EBBS Subscale Scores

The study sample predominantly consisted of females (80.9%), aged 31–40 years (38.3%), who had undergone sleeve gastrectomy (94.6%). Preoperatively, 54% of participants were classified as Class III obesity based on BMI, whereas postoperatively, 37.6% were categorized as overweight. The mean TWL was 33.50 ± 16.22 kg, mean %TWL was 29.29 ± 11.01, and mean %WLM was 95.06 ± 12.94. Mean subscale scores of the EBBS were 4.93 ± 1.01 for food consumption, 3.25 ± 1.42 for general behaviors, 5.32 ± 0.92 for drink consumption, and 2.46 ± 0.98 for lifestyle (Table 1).

**Table 1:** Descriptive characteristics of the participants’ (*N* = 298)

Variables	Category	*n*	%
**Gender**	Female	241	80.9
	Male	57	19.1
**Age (year)**	20–30	69	23.1
	31–40	114	38.3
	41–50	80	26.8
	51–100	35	11.7
**Time since undergoing bariatric surgery**	< 6 months	191	64.1
	6 months – 1 year	65	21.9
	1–3 years	32	10.7
	3–5 years	10	3.3
**Surgery type**	Sleeve gastrectomy	282	94.6
	By-pass surgery	16	5.4
**BMI (pre‑surgery) (kg/m** ^**2**^ **)**	Overweight	1	0.3
	Obesity class I	41	13.8
	Obesity class II	95	31.9
	Obesity class III	161	54.0
**BMI (post‑surgery) (kg/m** ^**2**^ **)**	Normal weight	76	25.5
	Overweight	112	37.6
	Obesity class I	66	22.1
	Obesity class II	32	10.7
	Obesity class III	12	4.0
	**Minimum**	**Maximum**	**Mean ± SD**
**Weight-related outcomes**			
Height (m)	146	192	164.63 ± 8.42
Pre-surgery weight (kg)	74	198	112.53 ± 23.12
Current weight (kg)	47	165	79.03 ± 18.00
Minimum weight (kg)	47	162	77.10 ± 17.67
Total weight loss (kg)	0	85	33.50 ± 16.22
Total weight loss (%TWL)	0	55	29.29 ± 11.01
Excess weight loss (%EWL)	0	191	80.03 ± 34.32
Weight loss maintained (%WLM)	0	102	95.06 ± 12.94
Recovery from minimum weight (kg)	0	98	2.41 ± 8.03
**Eating behaviors for participants after surgery (Domains)**			
Food consumption	0	6	4.93 ± 1.01
Drink consumption	0	6	5.32 ± 0.92
General behaviors	0	6	3.25 ± 1.42
Lifestyle	0	4	2.46 ± 0.98

Note: *BMI* classification follows *WHO* cut-points Normal (18.5–24.9), Overweight (25.0–29.9), Obesity Class I (30.0–34.9), Class II (35.0–39.9), Class III (≥ 40.0), % *TWL*, percent total weight loss, % *EWL*, percent excess weight loss, % *WLM*, percent weight loss maintenance, *BMI*, body mass index, *SD*, standard deviation

### Distribution of Responses Across EBBS Subscale Items

In the food consumption domain, the majority of participants reported consuming three main meals plus 2–3 snacks per day (53.7%). Participants who consumed sweets more than three times per week accounted for 40.9%, whereas those consuming sweets 0–1 times per week comprised 19.8%. Regarding vegetable intake, more than half of the participants reported consuming moderate portions (54.7%). In the drink consumption subscale, a large proportion of participants did not drink water during meals (67.4%). Non-consumers of alcohol represented 90.6%, and those who did not consume carbonated beverages accounted for 86.2%. In the general behaviors domain, a considerable portion of participants reported consuming their main meals within 10–15 min (40.9%). 54.7% described their pre-meal hunger as “moderate,” while 67.4% reported post-meal satiety as “high.” For the lifestyle domain, the most frequently reported physical activity pattern was 2–3 times per week for 30–60 min (46.3%). Most participants reported weighing themselves once per week (68.1%). The highest adherence rate was observed in the drink consumption subscale (88.7%), followed by food consumption (82.2%). The lowest adherence was in the general behaviors subscale (54.2%), with lifestyle showing moderate adherence (61.5%) (Table 2).

**Table 2:** The EBBS scale adherence information (*N* = 298)

Domains	*n* (%)
Food consumption	
Q1) How many meals do you eat per day?	
3 main meals + 4–6 snacks	63 (21.1)
3 times (breakfast, lunch, and dinner)	75 (25.2)
3 main meals + 2–3 snacks	160 (53.7)
Q4) How many times a week do you eat sweets? (Excluding breakfasts)	
More than 3 times/week	122 (40.9)
2 times/week	117 (39.3)
0–1 time/week	59 (19.8)
Q9) How is the portion of vegetables you consume during the meal?	
Large	46 (15.4)
Medium	163 (54.7)
Small	89 (29.9)
**Drink consumption**	
Q3) How much water do you drink during the meal?	
More than 2 glasses	17 (5.7)
1 glass	80 (26.8)
None	201 (67.4)
Q5) How many times a week do you drink wine or alcohol?	
More than 2 times/week	5 (1.7)
1 time/week	23 (7.7)
Never	270 (90.6)
Q6) How many times a week do you drink sparkling beverages?	
More than 2 times/week	14 (4.7)
1 time/week	27 (9.1)
Never	257 (86.2)
**General behaviors**	
Q2) How quickly do you consume the main meals?	
10–15 min	122 (40.9)
15–20 min	117 (39.3)
More than 25 min	59 (19.8)
Q7) How hungry do you feel before meals?	
Elevated	46 (15.4)
Medium	163 (54.7)
Scant	89 (29.9)
Q8) How full do you feel after meals?	
Scant	17 (5.7)
Medium	80 (26.8)
Elevated	201 (67.4)
**Lifestyle**	
Q10) How much time do you spend on physical activity?	
0–1 time/week 30–60 min	104 (34.9)
2–3 times/week 30–60 min	138 (46.3)
5 times/week 30–60 min	56 (18.8)
Q11) How often do you weigh yourself?	
Never	17 (5.7)
1 time/month	78 (26.2)
1 time/week	203 (68.1)

### Associations Between EBBS Subscales and Weight-Related Variables

When examining the relationships between EBBS subscales and weight-related variables, negative significant correlations were observed between preoperative weight and the food consumption and general behaviors subscales (*r*=-0.131 and *r*=-0.180, respectively). TWL (kg) and %TWL were also negatively and significantly correlated with the food consumption, drink consumption, and general behaviors subscales. No significant correlations were found between %WLM or recovery from minimum weight (kg) and any of the EBBS subscales (Table 3).

**Table 3:** Correlation between weight-related outcomes and EBBS domains

Variables	Food consumption	Drink consumption	General behaviors	Lifestyle
Pre-surgery weight (kg)	− 0.131*	− 0.063	− 0.180**	0.112
Total weight lost (kg)	− 0.254**	− 0.275**	− 0.274**	0.033
Total weight lost (%)	− 0.227**	− 0.305**	− 0.219**	− 0.050
Excess weight loss (%)	− 0.132*	− 0.293**	− 0.199**	− 0.140*
Weight loss maintained (%)	0.008	0.011	0.033	0.075
Recovery from minimum weight (kg)	− 0.010	− 0.032	− 0.068	− 0.111

r Correlation coefficient, p Significance value *<0.05 **<0.01 (Pearson correlation 2-tailed)

### Weight Loss Maintenance

The greatest weight loss occurred in males aged 31–40 years and females aged 20–30 years. 53% of participants experienced ≥ 29%TWL, while 47% experienced < 29. The majority of participants (76.2%) maintained ≥ 95% of their weight loss, whereas 23.8% maintained less than 95%. Most participants (81.5%) gained 0–2.41 kg postoperatively, while only 18.5% gained 2.42 kg or more.

### Comparison of EBBS Domain Scores According to Time Elapsed After Surgery

Comparison of EBBS scores based on postoperative follow-up duration revealed statistically significant differences in the drink consumption and general behaviors subscales. General behaviors scores were highest in the first 6 months postoperatively (3.47 ± 1.27) and significantly declined to 2.60 ± 1.74 from the 13th month onwards (F = 7.60, *p* = 0.001). Similarly, drink consumption adherence scores were significantly higher in the early period (5.52 ± 0.73) compared to the mid-term and late periods (F = 13.90, *p* < 0.001). No significant time-dependent changes were observed for food consumption or lifestyle subscale scores (Table 4).

**Table 4:** Comparison of EBBS domain scores according to time elapsed after surgery

EBBS domains	Early postoperative (< 6 months) (*n* = 191) Mean ± SD	Mid-term (7–12 months) (*n* = 65) Mean ± SD	Late postoperative (≥ 13 months) (*n* = 42) Mean ± SD	F	*p*
**Food consumption**	4.97 ± 0.97	4.86 ± 1.01	4.86 ± 1.24	0.43	0.653
**Drink consumption**	5.52 ± 0.73	5.06 ± 1.06	4.83 ± 1.19	13.90	0.000*
**General behaviors**	3.47 ± 1.27	3.06 ± 1.50	2.60 ± 1.74	7.60	0.001*
**Lifestyle**	2.45 ± 0.97	2.62 ± 0.88	2.31 ± 1.18	1.32	0.268

*SD* Standard deviation, *F* one-way *ANOVA*, p Significance value, *p < 0.05

### Predictors of Postoperative %TWL

Hierarchical linear regression analyses were conducted separately for each EBBS subscale to examine their associations with %TWL before and after adjustment for clinical and demographic covariates. In the unadjusted models (Model 1), higher scores on Food consumption (B = − 2.457, β=−0.227, *p*<0.001), Drink consumption (B = − 2.613, β=−0.219, *p*<0.001), and General behaviors (B = − 2.357, β=−0.305, *p*<0.001) were significantly associated with lower %TWL. The Lifestyle subscale was not significantly associated with %TWL (*p* = 0.389). After adjustment for age, gender, time since surgery (< 6 months), surgery type (sleeve gastrectomy), and pre-surgery weight (Model 2), Food consumption (B = − 2.419, β=−0.224, *p*<0.001), Drink consumption (B = − 1.411, β=−0.118, *p* = 0.039), and General behaviors (B = − 1.663, β=−0.216, *p*<0.001) remained significantly associated with %TWL, whereas Lifestyle remained non-significant (*p* = 0.182). The magnitude and direction of the associations for Food consumption and General behaviors were largely preserved after adjustment. Across adjusted models, shorter time since bariatric surgery (< 6 months) was consistently associated with lower %TWL (all *p*<0.001), and higher pre-surgery weight was positively associated with %TWL (all *p*≤0.001). Age showed a modest negative association only in the Food consumption model (*p* = 0.013), and female gender was significant only in that model (*p* = 0.045). Surgery type was not independently associated with %TWL in any adjusted model. These findings indicate that specific EBBS behavioral domains particularly Food consumption and General behaviors demonstrate independent associations with %TWL beyond relevant clinical covariates (Tables 5 and 6).

**Table 5:** Hierarchical linear regression models predicting %TWL from EBBS subscales and selected covariates

Variables	B	SE	β	95%CI (L-U)	t	*p*
**Food consumption (Model 1)**	-2.457	0.612	-0.227	-3.660 - -1.253	-4.017	0.000*
**Food consumption (Model 2)**	-2.419	0.573	-0.224	-3.546 - -1.292	-4.224	0.000*
**Age**	-0.163	0.065	-0.134	-0.292 - -0.035	-2.499	0.013*
Gender (female)**	3.288	1.636	0.118	0.068–6.507	2.010	0.045*
**Time since undergoing bariatric surgery** (< 6 months)**	-8.145	1.199	-0.355	-10.504 - -5.786	-6.796	0.000*
Surgery type (sleeve gastrectomy)**	2.993	2.573	0.061	-2.070–8.057	1.163	0.246
**Pre-surgery weight**	0.091	0.028	0.190	0.035–0.146	3.226	0.001*
**Drink consumption (Model 1)**	-2.613	0.677	-0.219	-3.945 - -1.282	-3.862	0.000*
**Drink consumption (Model 2)**	-1.411	0.681	-0.118	-2.752 - -0.070	-2.071	0.039*
**Age**	-0.086	0.066	-0.071	-0.217–0.044	-1.301	0.194
Gender (female)**	3.318	1.705	0.119	-0.037–6.673	1.947	0.053
**Time since undergoing bariatric surgery** (< 6 months)**	-7.409	1.278	-0.323	-9.924 - -4.894	-5.797	0.000*
Surgery type (sleeve gastrectomy)**	4.688	2.638	0.096	-0.503–9.876	1.777	0.077
**Pre-surgery weight**	0.099	0.029	0.207	0.043–0.155	3.454	0.000*
**General behaviors (Model 1)**	-2.357	0.427	-0.305	-3.197 - -1.516	-5.517	0.000*
**General behaviors (Model 2)**	-1.663	0.412	-0.216	-2.474 - -0.852	-4.037	0.000*
**Age**	-0.062	0.065	-0.050	-0.190–0.067	-0.941	0.347
Gender (female)**	2.470	1.631	0.088	-0.740–5.679	1.515	0.131
**Time since undergoing bariatric surgery** (< 6 months)**	-7.150	1.227	-0.312	-9.565 - -4.735	-5.826	0.000*
Surgery type (sleeve gastrectomy)**	3.368	2.573	0.069	-1.695–8.431	1.309	0.192
**Pre-surgery weight**	0.096	0.028	0.202	0.041–0.152	3.437	0.000*
**Lifestyle (Model 1)**	-0.560	0.649	-0.050	-1.838–0.718	-0.863	0.389
**Lifestyle (Model 2)**	-0.795	0.593	-0.071	-1.962–0.373	-1.339	0.182
**Age**	-0.104	0.066	-0.085	-0.234–0.026	-1.573	0.117
Gender (female)**	2.372	1.676	0.085	-0.925–5.670	1.416	0.158
**Time since undergoing bariatric surgery** (< 6 months)**	-8.147	1.231	-0.355	-10.569 - -5.724	-6.618	0.000*
Surgery type (sleeve gastrectomy)**	4.202	2.633	0.086	-0.980–9.384	1.596	0.112
**Pre-surgery weight**	0.102	0.029	0.215	0.046–0.159	3.548	0.000*

*SE* Standard error, p Significance, *L* Lower bound, *U* Upper bound, *p < 0.05, **Dummy coding

**Table 6:** Model summary of hierarchical linear regression analyses predicting %TWL

EBBS Subscale	Model	*R* ^2^	Adj *R*^2^	ΔR²	F	*p*	Durbin-Watson	VIF
**Food consumption**	Model 1	0.052	0.048		16.135	< 0.001		
	Model 2	0.242	0.227	0.191	15.511	< 0.001	1.058	1.050–1.333
**Drink consumption**	Model 1	0.048	0.045		14.912	< 0.001		
	Model 2	0.208	0.191	0.160	12.702	< 0.001	1.051	1.073–1.365
**General behaviors**	Model 1	0.093	0.090		30.438	< 0.001		
	Model 2	0.239	0.223	0.145	15.193	< 0.001	1.131	1.063–1.326
**Lifestyle**	Model 1	0.003	-0.001		0.744	0.389		
	Model 2	0.201	0.184	0.198	12.186	< 0.001	1.019	1.025–1.331

p Significance, *VIF* Variance inflation factor

## Discussion

This study examined the relationship between postoperative eating- and lifestyle-related behaviors, as assessed by the Turkish version of the EBBS, and weight loss outcomes following MBS. In this study, higher EBBS subscale scores for food consumption, drink consumption, and general behaviors were consistently and negatively associated with %TWL, indicating that greater adherence scores were related to lower percentage total weight loss. These inverse associations remained statistically significant after adjustment for demographic and clinical covariates, particularly for food consumption and general behaviors, whereas the lifestyle subscale showed no significant relationship with %TWL. Similarly, TWL (kg) and %TWL demonstrated significant negative correlations with the same three behavioral domains, while no significant associations were observed with %WLM or recovery from minimum weight. Additionally, adherence to drink consumption and general behaviors declined significantly over time after surgery, and shorter postoperative duration was independently associated with lower %TWL, whereas higher preoperative weight was positively associated with %TWL.

In this study, the highest adherence was observed in the drink consumption subscale, while the lowest adherence was in the general behaviors subscale. High adherence in the drink consumption domain suggests that key recommendations, such as avoiding fluid intake during meals and minimizing carbonated beverages, are effectively implemented in clinical practice. These recommendations are consistent with postoperative guidance regarding separation of liquids and solids and restriction of carbonation. Although the evidence base is not entirely derived from randomized trials, these behaviors are recognized as contributing to smooth dietary progression and symptom management [17, 18].

Relatively low adherence in the general behaviors domain (e.g., 40.9% of participants consuming main meals within 10–15 min) is clinically meaningful. Slow eating, adequate chewing, and monitoring satiety cues are fundamental components of bariatric nutrition. Consensus findings emphasize that habits such as slow eating and self-regulation support long-term sustainability [19]. These results suggest that targeted behavioral counseling may be necessary to improve adherence in this domain among individuals following MBS.

From a cultural perspective, the high rate of non-alcohol consumption is noteworthy. In the Arabic adaptation of the EBBS, the alcohol item was reportedly removed for cultural reasons [11]. In the Turkish version, however, the item was retained based on expert review, and a substantial floor effect was observed. This suggests that country and subcultural differences may influence item functioning, and that future studies could employ methods such as differential item functioning to examine cultural invariance. On the other hand, the avoidance of alcohol post-MBS may also reflect clinical recommendations, given its high caloric content. Literature indicates that excessive alcohol intake can negatively affect weight loss due to increased calorie consumption and may contribute to nutrient deficiencies, particularly of B vitamins [17]. Nevertheless, the high overall adherence in the drink consumption subscale and the expected pattern of correlations support the clinical relevance of the scale in this context. Conversely, the relatively high proportion of participants consuming sweets ≥ 3 times per week (40.9%) draws attention to patterns of high-energy and sugary foods associated with recurrent weight gain. Recent systematic reviews reiterate that post-bariatric dietary nonadherence (including high-calorie foods and drinks, sweets, and low protein intake) along with low physical activity, is linked to recurrent weight gain [8]. In this framework, improving adherence in the food and drink subdomains may strengthen the associations observed in this study between adherence and weight loss outcomes.

Patterns of weight loss by age and gender are generally consistent with the literature. Previous studies report greater short to medium-term weight loss among younger age groups [20]. In the present study, the highest weight loss was observed in males aged 31–40 years and females aged 20–30 years, which may be interpreted as a variation of this trend. However, the biological and sociocultural determinants of these differences, such as hormonal status, activity patterns, and caregiving responsibilities, warrant further detailed investigation.

An important finding of this study was that several EBBS subscale scores showed negative correlations with weight-related outcomes. This directionality should be interpreted with caution, as higher scores on self-reported behavioral measures do not necessarily equate to superior weight loss outcomes and may reflect complex behavioral patterns that are not linearly related to weight change. Previous studies have documented heterogeneous associations between postoperative eating behaviors and weight outcomes, including instances in which certain eating behavior domains were not uniformly predictive of better weight results at follow-up, suggesting that the relationship between eating behaviors and weight change is multifactorial and may vary across different behavioral constructs and time points. Finally, systematic literature reviews emphasize that behavioral, psychological, and lifestyle factors interact in complex ways to influence long-term weight maintenance after metabolic bariatric surgery rather than exhibiting simple uniform positive correlations [7, 8, 21]. The negative correlations between preoperative weight and the food consumption and general behaviors subscale scores may suggest that individuals with higher baseline weight report greater challenges related to eating behavior regulation. This finding underscores the potential need for more intensive behavioral support, particularly in the early postoperative period [8]. Furthermore, the identification of time elapsed since surgery as an independent predictor of %TWL in regression analyses aligns with the literature, suggesting that weight loss evolves over time and may be accompanied by partial weight gain [19].

76.2% of participants maintained high levels of weight loss (%WLM ≥ 95%), whereas only 23.8% maintained below 95%. This finding highlights the effectiveness of MBS in sustaining weight loss in the early to mid-term postoperative period, particularly when behavioral adherence is high. Literature reports that the ability to maintain weight loss varies between 40 and 80%, depending on procedure type, follow-up duration, and behavioral factors [22, 23]. The high %WLM observed in the current study may reflect relatively good adherence in food and drink consumption or the comparatively short postoperative follow-up period. However, lower adherence in the general behaviors and lifestyle subdomains suggests that this cohort could be at risk for recurrent weight gain over the long term [24]. Furthermore, the majority of participants (81.5%) regained less than 2.41 kg postoperatively, while 18.5% exceeded this threshold. These rates are lower than those reported in long-term recurrent weight gain literature [25–27], likely reflecting the relatively short postoperative period of the sample. Overall, the findings indicate that early- and mid-term weight loss can be largely maintained, but a subset of patients still demonstrates clinically meaningful weight gain. In this context, targeting behaviors related to food intake and low physical activity may be critical for preventing postoperative weight gain [28, 29].

Assessments based on postoperative follow-up duration reveal that EBBS general behavior and drink consumption adherence scores decline significantly over time, with a notable regression occurring from the 13th month onwards. This pattern can be attributed to the conclusion of the “honeymoon period” and the gradual return of appetite, phases frequently highlighted in bariatric literature where initial strict adherence to recommendations tends to diminish over time [30]. While the peak adherence observed in general behaviors during the first six months reflects maximal surgical motivation, critical self-regulation mechanisms such as controlling eating speed and monitoring satiety signals appear to weaken as the follow-up duration increases [10]. Similarly, the decline in beverage adherence scores during the mid- and late terms suggests that patients may begin to relax fundamental rules, such as avoiding fluid intake during meals or carbonated beverages, as they move further away from the surgery date [11]. The lack of significant time-dependent changes in the food consumption and lifestyle subscales may indicate that behavioral patterns in these domains are either more deeply ingrained or maintain a more stable course due to the inherent nature of surgical restrictions [30]. Consequently, the observed decline in behavioral and beverage adherence after the first year emphasizes the critical need for sustained multidisciplinary support during long-term clinical follow-up [10].

The identification of the food consumption subscale score as an independent predictor of %TWL in regression analysis is also consistent with the literature. Recent studies utilizing the EBBS have found that either the total scale score or specific subdomains can be useful in predicting postoperative weight loss patterns or the risk of recurrent weight gain [10, 11, 31]. Notably, a study demonstrating the clinical utility of the EBBS in predicting long-term recurrent weight gain after sleeve gastrectomy further supports the prognostic significance of behavioral adherence [31].

An important consideration in interpreting the present findings relates to the translation and measurement properties of the Turkish version of the EBBS. Although the EBBS has been previously developed and validated, the present study was not designed as a full psychometric validation study. Internal consistency estimates for the Turkish version were lower than expected, and factor analytic procedures could not be reliably performed. These findings suggest that the EBBS may not function as a psychometrically uniform construct across all cultural contexts. Nevertheless, the observed associations between EBBS subscale scores and postoperative weight-related outcomes provide clinically meaningful insights into how postoperative eating and lifestyle behaviors relate to weight trajectories in Turkish patients who have undergone MBS. Rather than serving as evidence of formal scale validity, these correlations should be interpreted as indicators of contextual and clinical relevance. Similar challenges in cross-cultural adaptations of questionnaires have been reported, emphasizing that behavioral constructs may manifest differently depending on sociocultural and dietary norms [11, 32]. Taken together, the present findings suggest that while the Turkish version of the EBBS may require further refinement and formal psychometric validation in larger samples, it may still offer value as a clinically oriented screening tool to explore postoperative behavioral patterns and their relationship with weight outcomes.

### Limitations

This study is strengthened by its use of the Turkish adaptation of the EBBS to delineate domain-specific behavioral patterns and relate it to multiple weight-related outcomes. However, several limitations should be noted. The reliance on self-reported data, a single-center sample heavily weighted toward one procedure (sleeve gastrectomy 94.6%), and temporal heterogeneity in postoperative follow-up may introduce potential biases. In addition, the study was not designed as a full psychometric validation, and findings should therefore be interpreted primarily in terms of clinical relevance rather than formal scale validity.

## Conclusion

The findings of this study indicate that higher adherence scores in the food consumption, drink consumption, and general behaviors domains were independently and negatively associated with %TWL after adjustment for demographic and clinical covariates. Notably, adherence to recommended drink consumption behaviors and eating practices tended to decline over time, highlighting the need for ongoing patient education and follow-up beyond the early postoperative period. Taken together, these findings show that the EBBS, when used as intended, provides a structured assessment of postoperative adherence and shows meaningful but negative associations with weight-related outcomes. By providing a structured assessment of eating, drinking, general, and lifestyle behaviors, the scale can inform clinicians in supporting sustainable weight management after MBS. Given these characteristics, it should be considered a useful measurement tool in the clinical practice.

Although further psychometric refinement of the Turkish version is warranted, the scale may serve as a practical instrument in clinical practice to systematically monitor eating- and lifestyle-related behaviors after bariatric surgery. Future research should continue to refine the Turkish version of the EBBS and explore its utility in larger and more diverse patient populations. Given the potential cultural or environmental floor effects observed in certain items (e.g., alcohol), future refinements using differential item functioning and item response theory approaches are recommended. To further clarify the measurement properties and longitudinal clinical relevance of the Turkish EBBS, prospective cohort studies and repeated EBBS measurements would be valuable for modeling the dynamic relationships between behaviors and weight over time.

## Data Availability

No datasets were generated or analysed during the current study.
